# More Room

**Published:** 1874-03

**Authors:** 


More Room! More Room!!—Yes, we wish we had more space—many more pages to present our readers each month, filled with the choice thoughts of our Editorial Staff, Medical friends, and the many, many clinical facts that slip by without finding room in our columns.

We desire to enlarge the Record, to make it the *best* book of reference of current medical, practical, facts in the country. Twelve numbers containing condensed extracts as numerous as those found in the present, would, when enlarged, be of the greatest value to our friends. Can this not be done? Easily. Let each subscriber send us but one additional name and it will be accomplished.

